# Supplementary material for: Targeted induction of a silent fungal gene cluster encoding the bacteria-specific germination inhibitor fumigermin
Source: eLife. 2020 Feb 21;9:e52541. doi: 10.7554/eLife.52541 (PMC7034978; doi:10.7554/eLife.52541)
Supplement: Supplementary file 1. — Domain analysis using the CD-search tool (NCBI, Marchler-Bauer et al., 2017) of each of the cluster genes (shown in black and green), as well as the 5’ and 3’ neighboring genes. Based on similarity to known genes, the potential function of the encoded proteins was predicted. Gene sequences were downloaded from the FungiDB database (https://fungidb.org/fungidb/). [file elife-52541-supp1.pptx]

## Slide 1
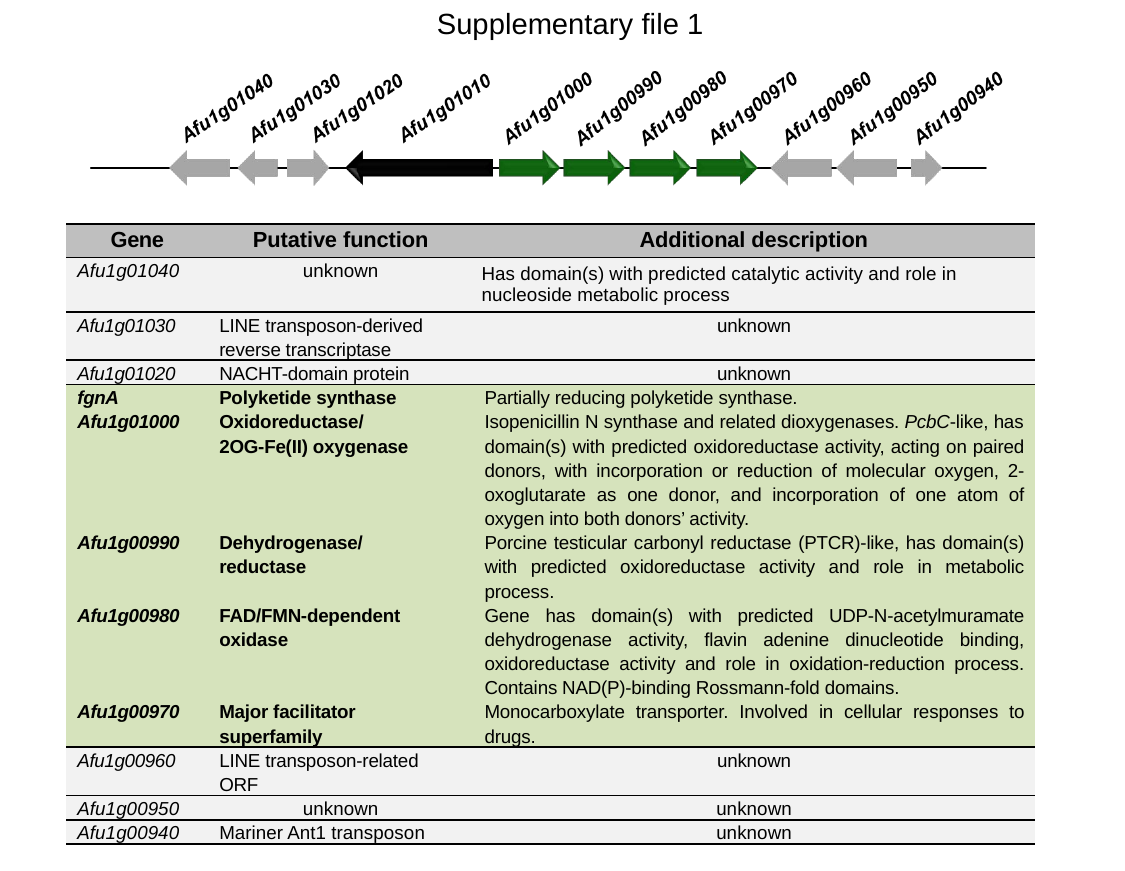

Supplementary file 1
| Gene | Putative function | Additional description |
| --- | --- | --- |
| Afu1g01040 | unknown | Has domain(s) with predicted catalytic activity and role in nucleoside metabolic process |
| Afu1g01030 | LINE transposon-derived reverse transcriptase | unknown |
| Afu1g01020 | NACHT-domain protein | unknown |
| fgnA | Polyketide synthase | Partially reducing polyketide synthase. |
| Afu1g01000 | Oxidoreductase/ 2OG-Fe(II) oxygenase | Isopenicillin N synthase and related dioxygenases. PcbC-like, has domain(s) with predicted oxidoreductase activity, acting on paired donors, with incorporation or reduction of molecular oxygen, 2-oxoglutarate as one donor, and incorporation of one atom of oxygen into both donors’ activity. |
| Afu1g00990 | Dehydrogenase/ reductase | Porcine testicular carbonyl reductase (PTCR)-like, has domain(s) with predicted oxidoreductase activity and role in metabolic process. |
| Afu1g00980 | FAD/FMN-dependent oxidase | Gene has domain(s) with predicted UDP-N-acetylmuramate dehydrogenase activity, flavin adenine dinucleotide binding, oxidoreductase activity and role in oxidation-reduction process. Contains NAD(P)-binding Rossmann-fold domains. |
| Afu1g00970 | Major facilitator superfamily | Monocarboxylate transporter. Involved in cellular responses to drugs. |
| Afu1g00960 | LINE transposon-related ORF | unknown |
| Afu1g00950 | unknown | unknown |
| Afu1g00940 | Mariner Ant1 transposon | unknown |
