# Supplementary material for: Targeted induction of a silent fungal gene cluster encoding the bacteria-specific germination inhibitor fumigermin
Source: eLife. 2020 Feb 21;9:e52541. doi: 10.7554/eLife.52541 (PMC7034978; doi:10.7554/eLife.52541)
Supplement: Supplementary file 2. [file elife-52541-supp2.pptx]

## Slide 1
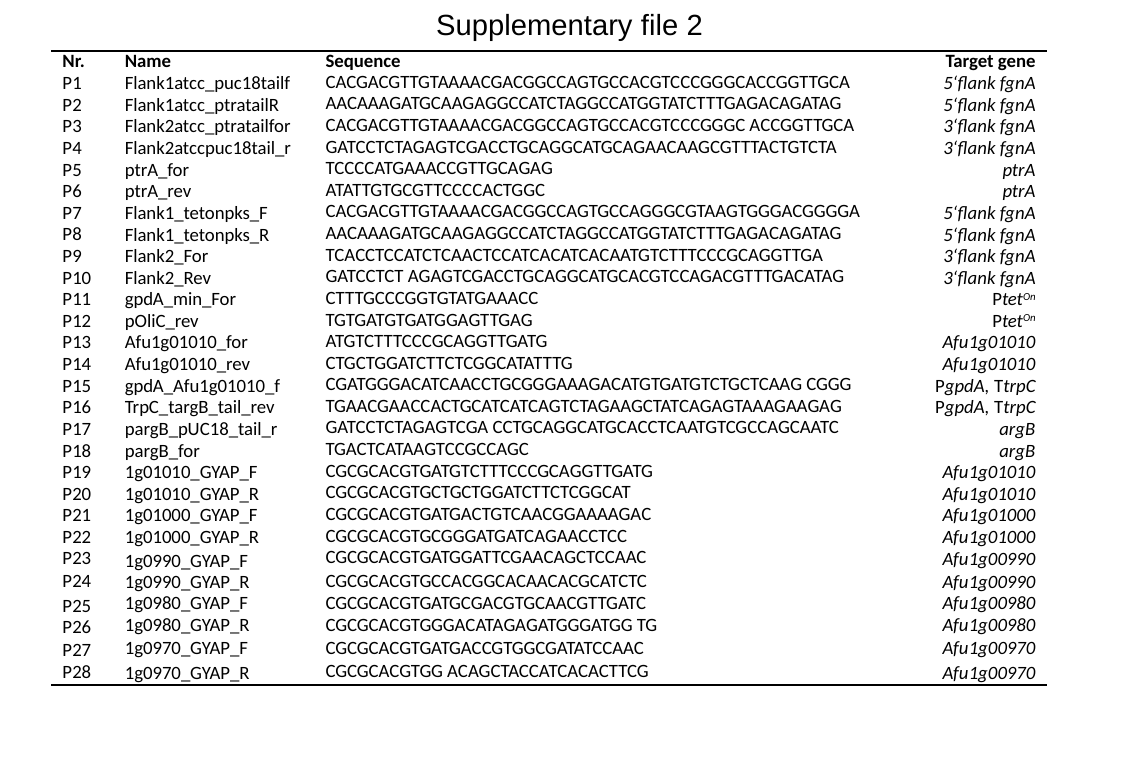

Supplementary file 2
| Nr. | Name | Sequence | Target gene |
| --- | --- | --- | --- |
| P1 | Flank1atcc\_puc18tailf | cacgacgttgtaaaacgacggccagtgccacgtcccgggcaccggttgca | 5‘flank fgnA |
| P2 | Flank1atcc\_ptratailR | aacaaagatgcaagaggccatctaggccatggtatctttgagacagatag | 5‘flank fgnA |
| P3 | Flank2atcc\_ptratailfor | cacgacgttgtaaaacgacggccagtgccacgtcccgggc accggttgca | 3‘flank fgnA |
| P4 | Flank2atccpuc18tail\_r | gatcctctagagtcgacctgcaggcatgcagaacaagcgtttactgtcta | 3‘flank fgnA |
| P5 | ptrA\_for | TCCCCATGAAACCGTTGCAGAG | ptrA |
| P6 | ptrA\_rev | ATATTGTGCGTTCCCCACTGGC | ptrA |
| P7 | Flank1\_tetonpks\_F | cacgacgttgtaaaacgacggccagtgccagggcgtaagtgggacgggga | 5‘flank fgnA |
| P8 | Flank1\_tetonpks\_R | aacaaagatgcaagaggccatctaggccatggtatctttgagacagatag | 5‘flank fgnA |
| P9 | Flank2\_For | tcacctccatctcaactccatcacatcacaatgtctttcccgcaggttga | 3‘flank fgnA |
| P10 | Flank2\_Rev | gatcctct agagtcgacctgcaggcatgcacgtccagacgtttgacatag | 3‘flank fgnA |
| P11 | gpdA\_min\_For | ctttgcccggtgtatgaaacc | PtetOn |
| P12 | pOliC\_rev | Tgtgatgtgatggagttgag | PtetOn |
| P13 | Afu1g01010\_for | Atgtctttcccgcaggttgatg | Afu1g01010 |
| P14 | Afu1g01010\_rev | Ctgctggatcttctcggcatatttg | Afu1g01010 |
| P15 | gpdA\_Afu1g01010\_f | cgatgggacatcaacctgcgggaaagacatgtgatgtctgctcaag cggg | PgpdA, TtrpC |
| P16 | TrpC\_targB\_tail\_rev | Tgaacgaaccactgcatcatcagtctagaagctatcagagtaaagaagag | PgpdA, TtrpC |
| P17 | pargB\_pUC18\_tail\_r | gatcctctagagtcga cctgcaggcatgcacctcaatgtcgccagcaatc | argB |
| P18 | pargB\_for | tgactcataagtccgccagc | argB |
| P19 | 1g01010\_GYAP\_F | Cgcgcacgtgatgtctttcccgcaggttgatg | Afu1g01010 |
| P20 | 1g01010\_GYAP\_R | Cgcgcacgtgctgctggatcttctcggcat | Afu1g01010 |
| P21 | 1g01000\_GYAP\_F | Cgcgcacgtgatgactgtcaacggaaaagac | Afu1g01000 |
| P22 | 1g01000\_GYAP\_R | Cgcgcacgtgcgggatgatcagaacctcc | Afu1g01000 |
| P23 | 1g0990\_GYAP\_F 1g0990\_GYAP\_R | Cgcgcacgtgatggattcgaacagctccaac | Afu1g00990 |
| | | Cgcgcacgtgccacggcacaacacgcatctc | Afu1g00990 |
| P24 | | | |
| P25 | 1g0980\_GYAP\_F | Cgcgcacgtgatgcgacgtgcaacgttgatc | Afu1g00980 |
| | 1g0980\_GYAP\_R | cgcgcacgtgggacatagagatgggatgg tg | Afu1g00980 |
| P26 | | | |
| | 1g0970\_GYAP\_F | Cgcgcacgtgatgaccgtggcgatatccaac | Afu1g00970 |
| P27 | | | |
| | 1g0970\_GYAP\_R | cgcgcacgtgg acagctaccatcacacttcg | Afu1g00970 |
| P28 | | | |
