# Supplementary material for: Targeted induction of a silent fungal gene cluster encoding the bacteria-specific germination inhibitor fumigermin
Source: eLife. 2020 Feb 21;9:e52541. doi: 10.7554/eLife.52541 (PMC7034978; doi:10.7554/eLife.52541)
Supplement: Supplementary file 3. [file elife-52541-supp3.pptx]

## Slide 1
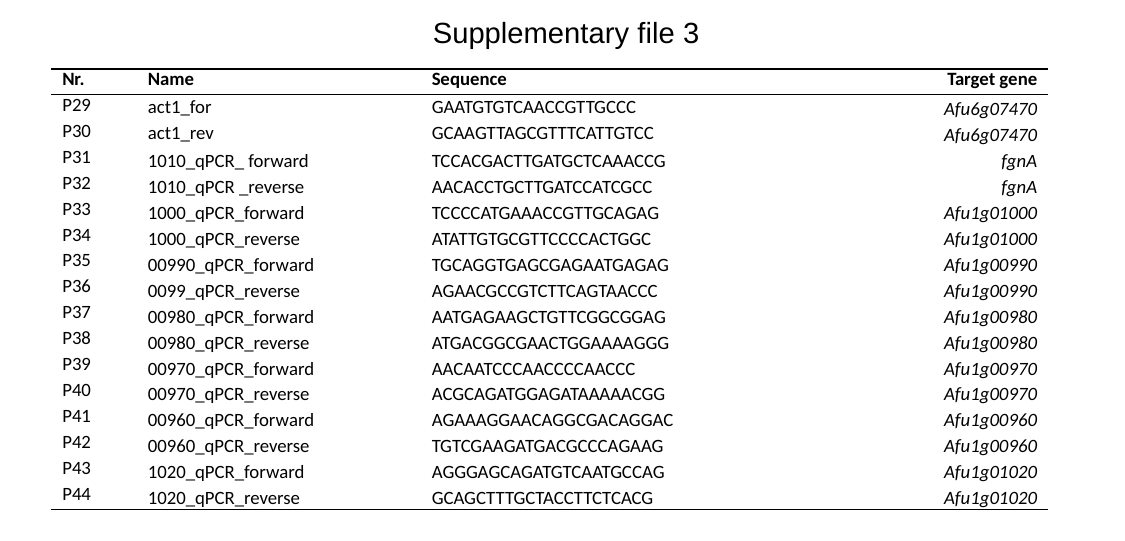

Supplementary file 3
| Nr. | Name | Sequence | Target gene |
| --- | --- | --- | --- |
| P29 | act1\_for | GAATGTGTCAACCGTTGCCC | Afu6g07470 |
| P30 | act1\_rev | GCAAGTTAGCGTTTCATTGTCC | Afu6g07470 |
| P31 | 1010\_qPCR\_ forward | TCCACGACTTGATGCTCAAACCG | fgnA |
| P32 | 1010\_qPCR \_reverse | AACACCTGCTTGATCCATCGCC | fgnA |
| P33 | 1000\_qPCR\_forward | TCCCCATGAAACCGTTGCAGAG | Afu1g01000 |
| P34 | 1000\_qPCR\_reverse | ATATTGTGCGTTCCCCACTGGC | Afu1g01000 |
| P35 | 00990\_qPCR\_forward | TGCAGGTGAGCGAGAATGAGAG | Afu1g00990 |
| P36 | 0099\_qPCR\_reverse | AGAACGCCGTCTTCAGTAACCC | Afu1g00990 |
| P37 | 00980\_qPCR\_forward | AATGAGAAGCTGTTCGGCGGAG | Afu1g00980 |
| P38 | 00980\_qPCR\_reverse | ATGACGGCGAACTGGAAAAGGG | Afu1g00980 |
| P39 | 00970\_qPCR\_forward | AACAATCCCAACCCCAACCC | Afu1g00970 |
| P40 | 00970\_qPCR\_reverse | ACGCAGATGGAGATAAAAACGG | Afu1g00970 |
| P41 | 00960\_qPCR\_forward | AGAAAGGAACAGGCGACAGGAC | Afu1g00960 |
| P42 | 00960\_qPCR\_reverse | TGTCGAAGATGACGCCCAGAAG | Afu1g00960 |
| P43 | 1020\_qPCR\_forward | AGGGAGCAGATGTCAATGCCAG | Afu1g01020 |
| P44 | 1020\_qPCR\_reverse | GCAGCTTTGCTACCTTCTCACG | Afu1g01020 |
