# Supplementary material for: Targeted induction of a silent fungal gene cluster encoding the bacteria-specific germination inhibitor fumigermin
Source: eLife. 2020 Feb 21;9:e52541. doi: 10.7554/eLife.52541 (PMC7034978; doi:10.7554/eLife.52541)
Supplement: Supplementary file 4. [file elife-52541-supp4.pptx]

## Slide 1
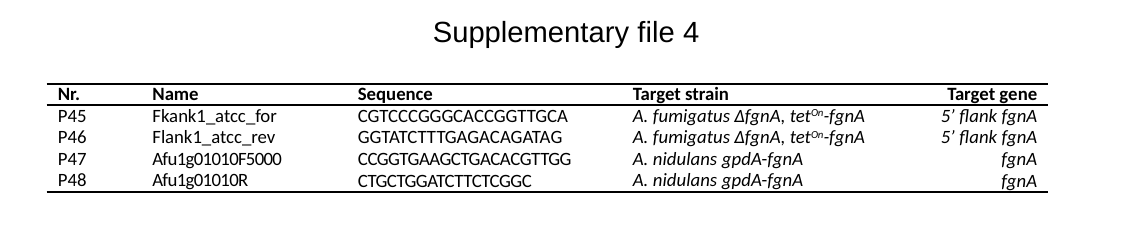

Supplementary file 4
| Nr. | Name | Sequence | Target strain | Target gene |
| --- | --- | --- | --- | --- |
| P45 | Fkank1\_atcc\_for | cgtcccgggcaccggttgca | A. fumigatus ΔfgnA, tetOn-fgnA | 5’ flank fgnA |
| P46 | Flank1\_atcc\_rev | ggtatctttgagacagatag | A. fumigatus ΔfgnA, tetOn-fgnA | 5’ flank fgnA |
| P47 | Afu1g01010F5000 | CCGGTGAAGCTGACACGTTGG | A. nidulans gpdA-fgnA | fgnA |
| P48 | Afu1g01010R | CTGCTGGATCTTCTCGGC | A. nidulans gpdA-fgnA | fgnA |
