# Supplementary material for: Targeted induction of a silent fungal gene cluster encoding the bacteria-specific germination inhibitor fumigermin
Source: eLife. 2020 Feb 21;9:e52541. doi: 10.7554/eLife.52541 (PMC7034978; doi:10.7554/eLife.52541)
Supplement: Supplementary file 5. [file elife-52541-supp5.pptx]

## Slide 1
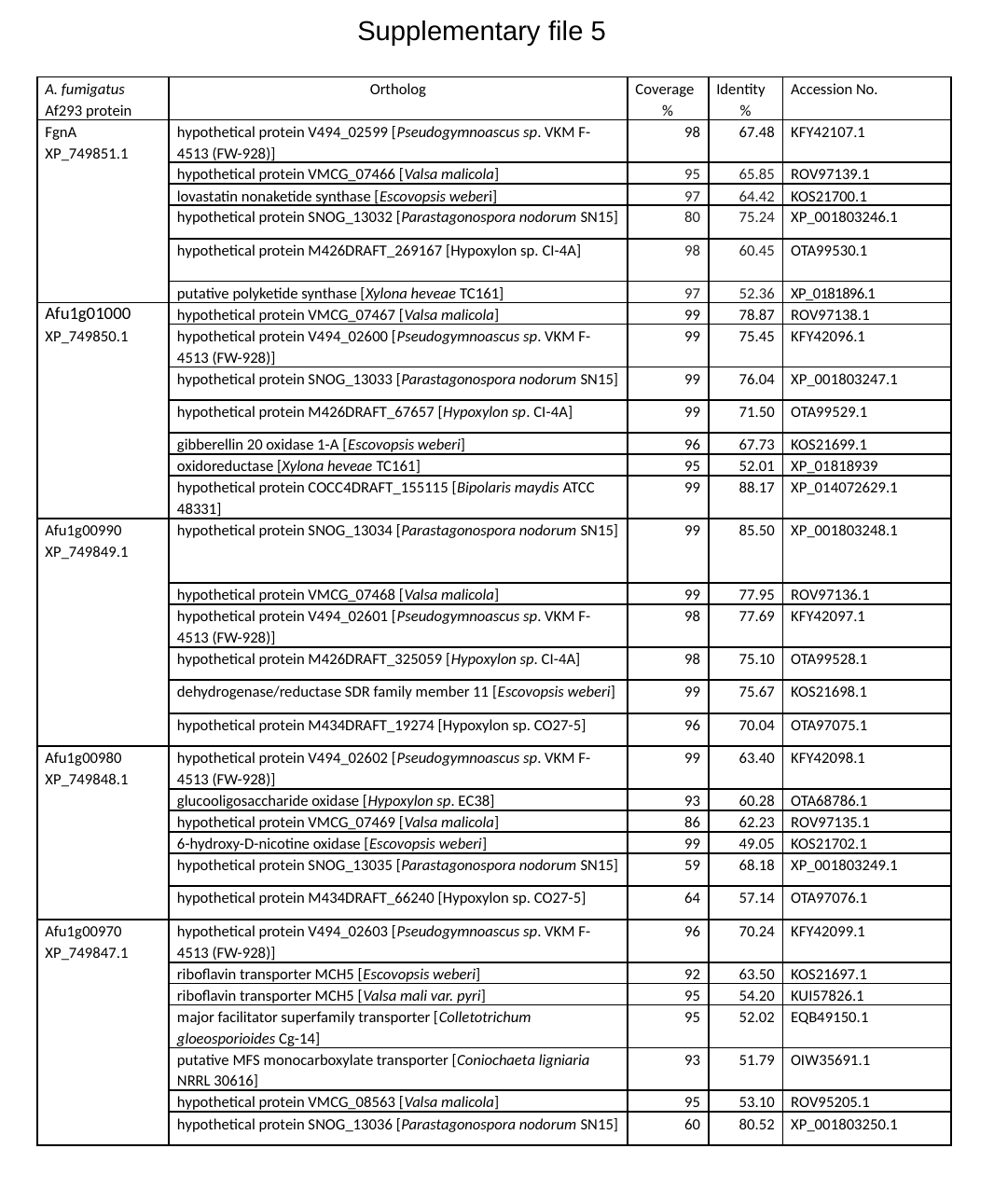

Supplementary file 5
| A. fumigatus Af293 protein | Ortholog | Coverage % | Identity % | Accession No. |
| --- | --- | --- | --- | --- |
| FgnA XP\_749851.1﻿ | hypothetical protein V494\_02599 [Pseudogymnoascus sp. VKM F-4513 (FW-928)] | 98 | 67.48 | KFY42107.1 |
| | hypothetical protein VMCG\_07466 [Valsa malicola] | 95 | 65.85 | ROV97139.1 |
| | lovastatin nonaketide synthase [Escovopsis weberi] | 97 | 64.42 | KOS21700.1 |
| | hypothetical protein SNOG\_13032 [Parastagonospora nodorum SN15] | 80 | 75.24 | XP\_001803246.1 |
| | hypothetical protein M426DRAFT\_269167 [Hypoxylon sp. CI-4A] | 98 | 60.45 | OTA99530.1 |
| | putative polyketide synthase [Xylona heveae TC161] | 97 | 52.36 | XP\_0181896.1 |
| Afu1g01000 | hypothetical protein VMCG\_07467 [Valsa malicola] | 99 | 78.87 | ROV97138.1 |
| XP\_749850.1 | hypothetical protein V494\_02600 [Pseudogymnoascus sp. VKM F-4513 (FW-928)] | 99 | 75.45 | KFY42096.1 |
| | hypothetical protein SNOG\_13033 [Parastagonospora nodorum SN15] | 99 | 76.04 | XP\_001803247.1 |
| | hypothetical protein M426DRAFT\_67657 [Hypoxylon sp. CI-4A] | 99 | 71.50 | OTA99529.1 |
| | gibberellin 20 oxidase 1-A [Escovopsis weberi] | 96 | 67.73 | KOS21699.1 |
| | oxidoreductase [Xylona heveae TC161] | 95 | 52.01 | XP\_01818939 |
| | hypothetical protein COCC4DRAFT\_155115 [Bipolaris maydis ATCC 48331] | 99 | 88.17 | XP\_014072629.1 |
| Afu1g00990 XP\_749849.1 | hypothetical protein SNOG\_13034 [Parastagonospora nodorum SN15] | 99 | 85.50 | XP\_001803248.1 |
| | hypothetical protein VMCG\_07468 [Valsa malicola] | 99 | 77.95 | ROV97136.1 |
| | hypothetical protein V494\_02601 [Pseudogymnoascus sp. VKM F-4513 (FW-928)] | 98 | 77.69 | KFY42097.1 |
| | hypothetical protein M426DRAFT\_325059 [Hypoxylon sp. CI-4A] | 98 | 75.10 | OTA99528.1 |
| | dehydrogenase/reductase SDR family member 11 [Escovopsis weberi] | 99 | 75.67 | KOS21698.1 |
| | hypothetical protein M434DRAFT\_19274 [Hypoxylon sp. CO27-5] | 96 | 70.04 | OTA97075.1 |
| Afu1g00980 XP\_749848.1 | hypothetical protein V494\_02602 [Pseudogymnoascus sp. VKM F-4513 (FW-928)] | 99 | 63.40 | KFY42098.1 |
| | glucooligosaccharide oxidase [Hypoxylon sp. EC38] | 93 | 60.28 | OTA68786.1 |
| | hypothetical protein VMCG\_07469 [Valsa malicola] | 86 | 62.23 | ROV97135.1 |
| | 6-hydroxy-D-nicotine oxidase [Escovopsis weberi] | 99 | 49.05 | KOS21702.1 |
| | hypothetical protein SNOG\_13035 [Parastagonospora nodorum SN15] | 59 | 68.18 | XP\_001803249.1 |
| | hypothetical protein M434DRAFT\_66240 [Hypoxylon sp. CO27-5] | 64 | 57.14 | OTA97076.1 |
| Afu1g00970 XP\_749847.1 | hypothetical protein V494\_02603 [Pseudogymnoascus sp. VKM F-4513 (FW-928)] | 96 | 70.24 | KFY42099.1 |
| | riboflavin transporter MCH5 [Escovopsis weberi] | 92 | 63.50 | KOS21697.1 |
| | riboflavin transporter MCH5 [Valsa mali var. pyri] | 95 | 54.20 | KUI57826.1 |
| | major facilitator superfamily transporter [Colletotrichum gloeosporioides Cg-14] | 95 | 52.02 | EQB49150.1 |
| | putative MFS monocarboxylate transporter [Coniochaeta ligniaria NRRL 30616] | 93 | 51.79 | OIW35691.1 |
| | hypothetical protein VMCG\_08563 [Valsa malicola] | 95 | 53.10 | ROV95205.1 |
| | hypothetical protein SNOG\_13036 [Parastagonospora nodorum SN15] | 60 | 80.52 | XP\_001803250.1 |
